# Behavioral versus Biological Definitions of Dementia Symptoms: Recognizing that Worthwhile Interventions already Exist

**DOI:** 10.21926/obm.geriatr.1904079

**Published:** 2019-10-10

**Authors:** Robert J. Brent

**Affiliations:** Department of Economics, Fordham University, Bronx, New York, USA

**Keywords:** Symptoms, behavior, interventions, effectiveness, costs, benefits, prevalence

## Abstract

**Background:**

The 2018 Alzheimer’s Disease Facts and Figures special report includes two new guidelines for measuring dementia symptoms. The first requires that a biomarker (biological factor) be added to a doctor’s clinical judgment of the cause of symptoms when determining whether dementia is present. The second involves identifying four stages of dementia: normal cognition, preclinical, MCI and dementia. Now only those with defining brain pathologies and significant symptoms will be judged to be persons with stage 4 dementia. This article examines the implications of adopting these two new guidelines. The implications are in terms of whether worthwhile dementia interventions can be said to exist, and the extent to which symptoms have to change for an intervention to be judged to have reduced the prevalence of dementia.

**Methods:**

A cost-benefit framework is used to examine the implications of the new guidelines. To undertake a cost-benefit analysis (CBA) a measure of dementia symptoms change is required for any intervention to be judged effective. A behavioral measure of dementia symptoms is thought more useful than a biological one. The instrument that is recommended and explained is the clinical dementia rating (CDR) scale, which is measured on a 0-to-18 interval. Using this instrument, three CBAs can be shown to exist, and from a contracted version of the CDR, estimates of the prevalence rates for the four stages of dementia are derived. The implications for future dementia research of using the full CDR instrument is presented in the discussion section.

**Results:**

The three CBAs that are reported and explained are years of education, Medicare eligibility and hearing aids. For each intervention, the analysis is in terms of demonstrating that it is effective, beneficial and socially worthwhile.

**Conclusions:**

By using a behavioral rather than a biological definition of dementia symptoms, we can show that worthwhile interventions already exist.

## 1. Introduction

In the 2018 Alzheimer’s Disease Facts and Figures special report [1] – hereafter “the Report” – they explain how the new guidelines in 2012 for measuring dementia differ from the old 1984 guidelines. In 1984, diagnostic criteria was based on a doctor’s clinical judgement of the cause of symptoms relying on reports from the individual, family members and friends, results of cognitive tests and general neurological assessments. The revised first guideline keep the same steps for diagnosis as before, but now add a requirement for a biomarker test. A biomarker is a biological factor that can be used to detect the presence of any disease. For Alzheimer’s disease (AD), the largest category of those classed with dementia, the biomarkers would be amyloid plaques and tau tangles.

For pharmacologic interventions, there may well be advantages in adopting the new guideline based on biological factors, so that one can ensure that those treatments that target a particular brain pathology go only to those with the particular brain pathology. Though, as the Report confirms [page 13]: “None of the pharmaceutical treatments (medications) available today for Alzheimer’s dementia slow or stop the damage and destruction of neurons that cause Alzheimer’s symptoms and make the disease fatal.” For non-pharmacologic interventions, such as exercise and cognitive stimulation, the verdict is the same. They also do not affect brain pathology. The implicit conclusion is that, if one is guided only by a biological definition of dementia, then there are no effective interventions for dementia that exist at this time.

However, if one uses a behavioral definition of dementia symptoms instead of a biological one, as we intend in this article, this negative conclusion is no longer applicable. For as the Report acknowledges, there are now many studies that show that exercise does have a positive effect on overall cognitive function and the rate of cognitive decline. The Report states that non-pharmacologic interventions are “beneficial” to people with AD. The defining characteristic of dementia is that it is a cognitive disease that interferes with the *activities* of daily living. If a person’s activities of daily living can continue and improve, and in this way benefits are provided, then interventions can be said to exist, even if the brain pathology is unaltered.

The purpose of this article is to show that, if one measures dementia symptoms using a behavioral instrument, then there are a whole new set of possible interventions that can be thought to exist. This is in contrast to using a biological measure, which would seem to indicate that there is very little that can be done today to reduce dementia. The behavioral instrument that we will be using is the Clinical Dementia Rating, CDR, scale (full name: *CDR® Dementia Staging Instrument* [2]).

Once one accepts a behavioral definition, which would focus an intervention on improving the ability of the person to carry on with the activities of daily living, this is equivalent to judging the effectiveness of an intervention by the benefits they provide and not the brain pathologies that it alters. When the benefits are expressed in monetary terms, they can be compared with the costs of the interventions to see if the difference is positive. If so, the intervention would not only be effective, it would also be socially worthwhile and therefore worth investing in. That is, the intervention would have passed a cost-benefit test [3, 4]. There already exists a number of interventions that have used a behavioral measure of dementia symptoms that have passed a cost-benefit test and we report on three of them. The plan is to recognize the existence of these interventions and to explain the nature of the benefits that have been generated by the interventions’ ability to reduce dementia symptoms. Since all three of the CBAs that we report on use the same behavioral dementia instrument based on the CDR, in the next section we provide an overview of the instrument.

## 2. Overview of the CDR Behavioral Symptoms Instrument

The CDR instrument is a well-accepted measure of dementia severity, that is used globally (available in 14 languages) and has been judged to be the best evidence based dementia scale in a recent review [2, 5, 6]. The CDR has six domains: memory, orientation, judgement and problem solving, community affairs, home and hobbies and personal care. Each of these domains is assessed on a 5 point scale regarding the extent of impairment (0 = none; 0.5 = questionable; 1 = mild; 2 = moderate and 3 = severe). The total score is called the CDR sum of boxes (SB) and has a range 0 to 18.

### 2.1 Frequency Distribution for the CDR Instrument

To help understand the scope for changing the CDR by dementia interventions, we present a summary of the instrument in the form of a frequency distribution. The data set we are going to use to assign frequencies to the 18 possible ratings comes from the National Alzheimer’s Coordinating Center (NACC). This data set was used to carry out all three of the CBAs that we will be reporting in the next section. The data set has been fully operational since September 2005. It has developed into a panel with the version used in our analysis spanning 13 years of visits by 35,183 individuals as of March 2017.

It is this latest version that we will use to construct the frequencies. These data consist of demographic, clinical, diagnostic, and neuropsychological information on participants with varying degrees of dementia severity, as well as those with normal cognition, who visited approximately 32 US Alzheimer’s Disease Centers (ADC). The data set is fully explained elsewhere [7–9]. Of the 35,183 individuals: 80% were white, 56% were female, 78% were eligible for Medicare, 13% had hearing aids, and the average number of years of education was 15.

We can see from Table 1 that the CDR-SB is a positively skewed distribution with a mode (0) less than the median (between 0.5 and 1) and the median less than the mean (2.87). A 0.5 change in the CDR-SB reduces the mean by 17%, but it would reduce the median by between 50% and 100%. In either case, for a typical older adult, a 0.5 change in the 18-point range in the CDR-SB by an intervention can be considered to be a non-inconsequential change.

### 2.2 Contracting the 18 Point CDR Instrument into 4 Classes

To provide further clarity into the meaning of a 0.5 change in the CDR-SB we can consider a simple contraction of the instrument into 4 classes, to be called stages. We have been led to consider this conversion because of the second changed guideline highlighted in the Report. There are now to be three stages of dementia, instead of just one, that is, having dementia. The three stages consist of two stages with symptoms – dementia disease itself and mild cognitive impairment (MCI) – and one stage without symptoms to be called preclinical dementia, to allow for the fact that brain changes may begin 20 years before symptoms occur. Logically, having no dementia can be regarded as a fourth stage to be added to the three stages with dementia. We can define these four stages in ascending order of dementia severity using the Report’s terminology and description for the three dementia stages (see Report page 15) as:

1. Normal cognition.
2. Pre-clinical (biomarkers that indicate the earliest signs of the disease, but people have no symptoms).
3. MCI (biomarker changes with cognitive decline that does not significantly affect activities of daily living).
4. Dementia (biomarker changes and behavioral symptoms).

Note that, for all three stages with dementia (stages 2 – 4), biomarkers must be present. Thus, the only characteristic that distinguishes the three dementia stages are the extent of symptoms that interfere with activities of daily living. Because the Report does not supply any measure of the extent of symptoms, one has no guidance as to how to distinguish no symptoms, from symptoms that are not significant, from symptoms that are significant. To help remedy this omission, we can consider the CDR instrument just presented to see how one can characterize symptom differences in order to be able to provide prevalence rates for the four classes. In what follows in this section, we provide an allocation of the prevalence rates for the four classes that would be implied if we assigned classes based on clear-cut correspondence to the six domains of the CDR instrument. In the discussion section, we provide alternative assignments of CDR frequencies for the four classes based on the prevalence rates that are presented in the Report itself.

Converting the 18-point CDR instrument in Table 1 to the four Report stages is straightforward for the first two stages. A CDR-SB score of 0 clearly corresponds to normal cognition and 36.22% of the sample can be assigned to this stage. We have seen earlier in our description of the 6 domains for the CDR that 0.5 = questionable. For an overall CDR-SB score to be 0.5, the clinician doing the appraisal has indicated that there is questionable impairment in one of the domains assessed in the CDR. Thus, a CDR-SB score of 0.5 can correspond to the pre-clinical dementia stage 2 in the Report. Based on Table 1, stage 2 would contain 10.75% of the sample.

If one did not assign class 2 to those with a CDR score of 0.5, and just lumped together classes 1 and 2, as they both strictly have no symptoms, then this would be negating the significance of symptoms, in contraction to the Report’s classes that specifies that symptoms matter in defining classes. All we are really doing to distinguish class 1 from class 2 is to state that for class 1 there are no symptoms, while for class 2 we are not sure if they have no symptoms (it is questionable). The only issue then is how to assign stages 3 and 4 to the 18-point CDR-SB scale.

A logical assignment of stages 3 and 4 (though in the discussion section we will also consider alternative empirical assignments coming from the Report) would be to consider a person to have MCI if they were judged to have detectable symptoms up to *one* of the CDR domains; but not one or more, which would denote stage 4 dementia. As we have seen, a CDR domain is rated on an interval of 0 to 3. Thus, more than 0.5, but not 3.0 or more, would be the MCI class. From Table 1 there is 10.79% in the CDR-SB range 1 – 1.5, and a further 7.58% in the range 2 – 2.5, resulting in 18.37% assigned to the stage 3 MCI class. That leaves 34.66% of the sample in the stage 4 dementia class. Table 2 shows the complete 4-stage CDR version.

In this abridged four class setting, a CDR-SB change of 0.5 would signify not only moving from class 1 to class 2, it would also signify moving from the preclinical to the MCI stage. In the latter context, it would take one 20% (0.05/2.5) of the way from moving from the preclinical to the MCI stage.

## 3. Three Dementia Interventions that Are Effective, Beneficial and Worth Financing

On the basis of an intervention being able to statistically significantly decrease the symptoms of dementia, as measured by the CDR-SB, it can be judged to be an effective intervention. In so far as the decrease in symptoms provides quantifiable increases in outcomes that are positive in monetary terms, an intervention can also be judged beneficial. When an intervention is able to provide benefits that exceed the total costs, it passes a cost-benefit test and becomes socially worthwhile to invest in. We now report some existing interventions that have been judged socially worthwhile [10–12]. All the interventions reported have used statistical methods supporting the notion that the interventions evaluated can be thought to be causally linked to outcomes and not just mere correlations. In particular, all the estimates came from equations that had numerous controls to account for socio-economic, demographic, and other health conditions. Most monetary valuations presented are expressed in 2010 prices.

### 3.1 Years of General Education

Under the brain reserve hypothesis, being educated gives the brain protective and compensating mechanisms making for greater resilience to the destructive pathologies of dementia [13]. Because brain size also determines dementia and leads to people favoring more education, it was important to control for hereditary factors in the estimation of the effects of years of education on the CDR-SB. as brain size runs in the family.

One year’s general education was found to have lowered the CDR-SB by 3.11 points [10]. Subsequent years of education also had positive effects (that is, symptoms declined), but at a declining rate. For example, the CDR-SB declined by 1.56 points for the second year of education and declined by 0.11 for the 28^th^ year of education (which was the maximum number of years of education in the NACC sample). For just 4 years of education the aggregate CDR-SB decline was 6.49 points and for the 28 year maximum the decline was 12.23. Because of these declines, there can be no doubt that providing a young person with years of education is an effective way of reducing dementia symptoms in the future. Table 1 showed that the maximum CDR-SB was 18.00. 4 years of education would cut the total by half and 28 years of education would reduce the total by two-thirds.

To show the benefits of education, one has to choose a quantifiable outcome measured in monetary terms. For the evaluation of years of education, the chosen outcome was the saving in caregiver costs by reducing the dementia symptoms. The more independent the living conditions for an older adult were from reducing the CDR-SB, the greater the caregiver cost savings. Since caregiving services has a market price, the cost savings could be readily valued. For example, any reduction in costs by a person no longer needing completely dependent care was valued at $28,501. Only a fraction of the people experiencing a reduction in dementia symptoms no longer needed complete dependent care, so the cost savings per point reduction in the CDR-SB were only a small part of $28,501. Nonetheless, the dementia benefits were positive, and amounted to $18,543 for 4 years of education and $41,787 for 28 years of education (once one allowed for the fact that the dementia benefits from education come well into the future and thus required discounting).

Taken on their own, the dementia benefits estimated are unlikely to cover all the costs of education. However, education has been found to have many other benefits, including financial, health and crime reducing benefits. The dementia benefits are therefore an important “other” category of benefits to be included in any education CBA.

### 3.2 Medicare Eligibility

Medicare eligibility has been shown to provide many health benefits [14, 15]. Lives were saved by Medicare’s provision of greater access to health services, causing older adults to make more visits to emergency departments for non-deferrable services. In the context of dementia, the greater access and utilization of services provided by Medicare eligibility led to a 0.92 reduction in the CDR-SB, making it an existing, effective intervention [11]. On the basis of Table 1 based on the 18 point rating, a 0.92 reduction would lower the mean rate of dementia symptoms by a third; and on the basis of Table 2, the 0.92 reduction would be enough to transform a person from being in the preclinical stage to end up being classed as having normal cognition.

To value the reduction in dementia symptoms from Medicare eligibility, basically the same outcome and valuation method was used as for the years of education intervention. That is, caregiver cost savings were used to value the benefits. The main difference between the Medicare eligibility and the years of education benefits method was that, although the same market prices were used for caregiver services, the proportion of people relying on dependent care living was lower for the Medicare eligibility intervention. This generated much larger monetary amounts for the 0.92 CDR-SB reduction than would have occurred if the 0.92 reduction had occurred through years of education. The reason why more people chose independent living, rather than costly dependent care living, was that for the Medicare eligibility evaluation, a quality of life (QoL) measure was inserted between the reduction in dementia and the choice of type of living location. The CDR-SB reduction increased the QoL of the older adult and this made them more likely to end up in independent living.

Using the caregiver cost saving method for valuing the independent living outcome generated by initial Medicare eligibility, the benefits were estimated to be $9,338. To see if these benefits exceed costs, a Medicare cost figure is required. The Medicare cost for a person for an individual aged 70+ was $10,904 if the person did not have dementia, and it was $17,444 if the person did have dementia. The difference between the two cost figures is $6,540. This is the additional cost that Medicare bears if older adults do not have their dementia symptoms reduced. The net-benefits of having Medicare eligibility is $2,798. This means that Medicare eligibility passed the cost-benefit test and is worth financing.

### 3.3 Hearing Aids

In the NACC data set, a hearing loss was defined as one where there is a functional impairment with hearing such that it reduced the ability to do daily activities, which fits in exactly with a behavioral definition of dementia symptoms. Examples of daily living listed were listening to the radio or television, or talking with family or friends. A hearing aid (HA) that restores normal hearing is going to be an intervention that has the ability to reduce dementia symptoms. In the actual HA evaluation, there were two categories of benefits, one for those with dementia symptoms and one for those without dementia symptoms. For the purposes of this article, we will refer only to the dementia symptoms part of the evaluation.

HAs, when controlling for all the usual factors (socio-economic, demographic and medical variables) and also the two interventions just covered (years of education and Medicare eligibility) lowered the CDR-SB by 0.73 points [12]. This is again higher than the 0.5 reduction that we explained in connection with Tables 1 and 2 to be a non-inconsequential change in dementia symptoms. An HA was found to be an existing, effective intervention.

A different benefits strategy than for the education and Medicare eligibility interventions was used to value the reduction in the CDR-SB from HAs. Instead of using the QoL as an intermediate step in the Medicare eligibility intervention, by being on the path to independent living, the QoL was used as the final outcome itself. It was because reducing a person’s dementia symptoms increases the person’s QoL that HAs were judged beneficial.

A Quality Adjusted Life Year (QALY) is often used in evaluations of health care interventions as the outcome measure [16]. This is the product of one’s life expectancy (life years, LY) and the quality of those years: QALY = LY × QoL. Any health care intervention one may imagine can be considered beneficial if it changes mortality and/or the quality of life of those years and thereby increase QALYs (ΔQALYs). For the HA evaluation, ΔQALYs over the 11 years of expected life years, and the 0.0023 change in the QoL, from the reduction in dementia symptoms was estimated to be 0.025 and this was the outcome magnitude.

In order to put a monetary value on the HA outcome to calculate the benefits, a price had to be put on a QALY. An extensive literature exists on how to set this price [17]. The price of a QALY chosen was $442,857. This figure was obtained by using a method derived from the value of a statistical life (VSL) of $65.089 million using a life expectancy of 23 years and a discount rate of 3% [18, 19]. Multiplying the QoL change of 0.025, by the price of a QALY of $442,857, and then multiplying the result by the 0.86 effectiveness ratio of hearing aids, generated the benefits valuation of $9,508.

The final step, to demonstrate that HAs were worth investing in, was to show that the benefits exceeded the costs. The lifetime costs of HAs, assuming that five sets would be required, were estimated to be $8,496. The HAs therefore produced net-benefits of $1,012 per person from the reduction in dementia symptoms. They passed the cost-benefit test.

## 4. Discussion

The three worthwhile interventions that have just been outlined were based on the behavioral measure of dementia symptoms contained in the 18-point CDR rating scale. We may not have known that worthwhile dementia interventions existed if we had not used the CDR measure. In this section, we will discuss the contribution that the use of the CDR may make in trying to estimate the prevalence of dementia in the US, and thereby may have some implications for future dementia research. It is important to understand that the NACC data do not constitute a population-based sample, so they are not appropriate for studying the incidence or prevalence of dementia at the population level (city, county, state, etc.) because of the varying sample strategies at each ADC center. Nonetheless, the NACC data set is a national one, and may be of some interest for indicating prevalence in the 4-stage dementia setting in the absence of a full set of estimates in the Report itself. The exercise may be useful provided that it is understood that any implications for policy must be very general and cannot be too specific.

The focal point is the Report’s citation of the ADAMS, the Ageing Demographics, and Memory Study, estimate of dementia being 13.9% of the population aged 71 and over [20]. Note that in the NACC data set of the 35,183 referred to in section 2, 41.2% were under the age of 71. They would be less likely to have dementia than in the ADAMS sample. The CDR would therefore always give a more conservative estimate of prevalence than that provided by ADAMS.

On the basis of the 4-class version of the 18-point CDR rating scale given in Table 2, the prevalence of dementia would be put at 34.66%; over twice the size of the ADAMS estimate. Table 2 was based on a definition of stage 3 that defined MCI as having up to one of the six dimensions that make up the CDR. As an alternative, let us define MCI as having the full interval for *two* of the six dimensions that make up the CDR. This involves redefining stage 3 to contain those with a CDR score between 1.0 and 6.0, which requires that stage 4 be defined to have a CDR score between 6.5 and 18.0. From Table 1 we see that 83.16% minus 46.97%, that is, 36.19% would be in stage 3. As a result, 16.84% would be in stage 4. Table 3 shows the resulting frequency distribution. The effect of the redefinition of stages 3 and 4 is to ensure that the ADAMS estimate of dementia prevalence is approximately obtained. However, this is at the expense of doubling the prevalence rate estimate for those with MCI. To estimate the MCI prevalence, the Report cites the report from the American Academy of Neurology [21] which obtained a 15.8% rate for people in the US aged 65 and over. This MCI prevalence rate is very much lower than the 36.19 estimate in Table 3.

The final possibility is to somewhat accept the Report’s prevalence rates for stages 3 and 4 (with 0.0 once more the definition of normal cognition for stage 1) and to alter the CDR rate for stage 2. This involves changing the CDR intervals for stages 2 and 3, again using the intervals and frequencies in Table 1. The resulting Table 4 would then be the frequency distribution most consistent with the Report’s cited prevalence rates using the CDR scale.

Although the Report does not give a prevalence rate for stage 2, it does cite a study that gives numbers of people in the preclinical stage (38.4 million) [22]. This is around three times larger than that cited for the MCI stage (11.6 million). This relative share would be somewhat comparable to the ratio of rates in stages 2 and 3 in Table 4. Thus, the Report would probably also endorse the 35.29% rate estimate for stage 2 in Table 4. This estimate is over three times that for stage 2 in Tables 2 and 3.

If Table 4 is interpreted to be the 4-stage frequency distribution for dementia prevalence, based on the 18-point CDR scale that is in the NACC data set, that is most consistent with some of the estimates cited in the Report, then for dementia research that priorities pharmaceutical interventions for stage 4 dementia, there are two main general implications. Firstly, because there are three times as many people with stage 2 as opposed to stage 4 dementia, priority should be given to finding medications that target those at the preclinical stage rather than the full dementia stage. This is especially true as the Report acknowledges that: “It is important to note that not all people with MCI or people who are in the proposed preclinical stage of Alzheimer’s will go on to develop Alzheimer’s dementia.”

Secondly, in so far as one is going to prioritize medications mainly for stage 4 dementia, there are inherent difficulties in just using the 4-stage categories as recommended in the new guidelines, rather than the 18-point CDR scale that is part of the NACC data set. It could be possible to achieve pioneering pharmaceutical breakthroughs, yet the numbers and rates of stage 4 dementia may not change, as we now illustrate.

Table 4 reveals that stage 4 dementia contains a wide range of CDR ratings, from 6.5 to 18.0, which is an 11.5-point interval. A new medication may be invented that reduces behavioral symptoms by 3 points, which is equivalent to removing one of the CDR dimensions completely. Alternatively, the new medication may reduce the behavioral symptoms by 6 points, which is equivalent to removing two CDR dimensions completely. In either case, if one were starting from a CDR score of 14 or more, then the prevalence rate of 13.56% would be unchanged. It would appear that the new breakthrough medications were completely unsuccessful. However, if one instead focuses on an 18-point CDR rating, then 3- and 6-point changes would be highly significant. This would be the case because the CDR-SB average was 2.87, and we have already reported on CBAs of interventions that are worth investing in, even with CDR changes of less than 1 point.

## 5. Summary and Conclusions

The 2018 Alzheimer’s Report uses the almost universal definition of dementia: “The characteristic symptoms of dementia are difficulties with memory, language, problem-solving and other cognitive skills that affect a person’s ability to perform everyday activities.” This is inherently a behavioral identification criterion for dementia. It is the performance of everyday activities that is important, not whether one has brain pathologies. The new guidelines that the Report endorses by requiring that there be a biological market in addition to a doctor’s clinical judgement can be questioned, as it is not central to what dementia inherently involves.

Brain pathologies may be correctable, but it needs to be shown that outcomes that affect daily living have been generated. Then these outcomes need to be valued in monetary terms in order to form benefits that can be compared to the monetary costs. It must not be forgotten that when successful future pharmaceutical have been developed, it may well be the case that they are very expensive to purchase. The costs of these interventions must not exceed the benefits in order for them to be worth funding.

Many effective, non-pharmaceutical, behavioral interventions have been identified in the literature. In a recent survey, the main ones were cardiovascular exercise guidelines, sleep hygiene strategies, and dietary modifications [23]. In addition, there is the whole field of cognitive rehabilitation that has evolved that has described successful interventions that are provided by occupational therapists, such as the Tailored Activity Program (TAP) [24]. What is now required is that all these non-pharmaceutical interventions be transformed into a cost-benefit framework so that their net-benefits can be established together with their proven effectiveness.

A main objective of this article was not only to point out the need for CBA to be included as a part of all evaluations of dementia interventions, but also to report on some actual CBAs that already exist. We do not need to wait for the pharmaceutical industry to come up with interventions that are worth funding; they are available now. Hearing loss affects millions of Americans and mirrors dementia symptoms. Yet the majority of those with hearing loss do not have hearing aids. Nursing homes need to ensure that hearing aids are provided and supported, and Medicare needs to cover hearing aids in its main plan. The benefits of hearing aids exceed their costs from reducing dementia in addition to all the other benefits they generate, which in total are thirty times greater than their costs. The benefit methodology used in the CBA relied on pricing QALYs. Every dementia intervention affects either life expectancy, or the quality of life, or both. Valuing QALYs is a general benefit methodology that is available to value benefits for any dementia evaluation.

The other two CBAs reported in this article, years of education and Medicare eligibility, are more long-term interventions. However, the benefit methodology used in these CBAs, involving cost savings, is an alternative that is also generally applicable. If dementia symptoms are reduced, older adults can reside in more independent living that does not require so much costly caregiver assistance. In fact, this cost-saving methodology is endorsed in the 2018 Alzheimer’s Report where there is a whole section on valuing the costs of dementia: “These projections suggest that a treatment that prevents, cures or slows down the progression of the disease could result in substantial savings to the US, health care system.” The Report estimates the lifetime cost of care for an individual living with dementia to be $341,840. Very large cost savings are potentially available.

All three CBAs reported used the CDR to measure the dementia symptoms. This a behavioral symptoms measure that does not depended on brain pathology. A whole section of this article was devoted to explaining this instrument. The six-dimension 18-point rating scale that comprises the CDR, that covers six behavioral domains, can be used to evaluate any dementia intervention.

We also showed how the 18-point rating scale could be used, very approximately, to estimate the US dementia prevalence rate using NACC data. To make this prevalence estimate, we explained the many ways how the full CDR instrument could be contracted to correspond with the second guideline change highlighted in the Report. This second guideline involved recognizing three stages of dementia, to which we aged a fourth stage, that is, no dementia. The end result was that there were four dementia stages, and for each one there could now be a prevalence figure, based on how one wished to allocate the numbers in the six CDR domains to the four stages.

For one allocation, the full dementia prevalence was much larger than the rate specified in the Report. For the allocation that corresponded more to prevalence rates for stages 3 and 4 given in the Report, the preclinical dementia stage 2 prevalence rate was tripled. No matter how reasonable the allocation formula, by using just four stages instead of an 18-point rating for dementia symptoms, the full dementia stage 4 would contain too wide a range of CDR intervals. This questioned the usefulness of the second guidelines highlighted in the Report. For it was likely that pharmaceutical interventions could greatly reduce dementia symptoms without reducing at all the measured prevalence rate estimating full dementia.

## Figures and Tables

**Table 1:** The frequency distribution for the 18-point CDR scale.

CDR-SB	Absolute Frequency	Relative Frequency %	Cumulative Frequency %

0.0	12,743	36.22	36.22
0.5	3,782	10.75	46.97
1.0 – 1.5	3,799	10.79	57.77
2.0 – 2.5	2,664	7.58	65.34
3.0 – 3.5	2,170	6.17	71.51
4.0 – 4.5	2,306	6.55	78.06
5.0 – 5.5	1,793	5.09	83.16
6.0 – 6.5	1,155	3.28	86.44
7.0 – 7.5	710	2.01	88.46
8.0 – 8.5	521	1.48	89.94
9.0 – 9.5	512	1.46	91.39
10.0 – 10.5	531	1.51	92.9
11.0 – 11.5	412	1.17	94.07
12.0 – 12.5	466	1.32	95.40
13.0 – 13.5	229	0.66	96.05
14.0	185	0.53	96.58
15.0	156	0.44	97.02
16.0	193	0.55	97.57
17.0	183	0.52	98.09
18.0	673	1.91	100.00
Total	35,183	100.00	

**Table 2:** The frequency distribution for the 4-stage CDR scale.

CDR-SB	Alzheimer Report Dementia Stage	Relative Frequency %	Cumulative Frequency %

0.0	1: Normal Cognition	36.22	36.22
0.5	2: Preclinical	10.75	46.97
1.0 – 2.5	3: MCI	18.37	65.34
3.0 – 18.0	4: Dementia	34.66	100.00

**Table 3:** The frequency distribution for the 4-stage CDR scale with stages 3 and 4 redefined.

CDR-SB	Alzheimer Report Dementia Stage	Relative Frequency %	Cumulative Frequency %

0.0	1: Normal Cognition	36.22	36.22
0.5	2: Preclinical	10.75	46.97
1.0 – 6.0	3: MCI	36.19	83.16
6.5 – 18.0	4: Dementia	16.84	100.00

**Table 4:** The frequency distribution for the 4-stage CDR scale with stages 2 and 3 redefined.

CDR-SB	Alzheimer Report Dementia Stage	Relative Frequency %	Cumulative Frequency %

0.0	1: Normal Cognition	36.22	36.22
0.5 – 3.5	2: Preclinical	35.29	71.51
4.0 – 6.0	3: MCIss	11.64	83.16
6.5 – 18.0	4: Dementia	16.84	100.00

## References

[1] Alzheimer’s Association. 2018 Alzheimer’s facts and figures. Alzheimers Dement. 2018; 14: 367–429.

[2] Morris JC. The clinical dementia rating (CDR). Current version and scoring rules. Neurology. 1993; 43: 2412–2414. 10.1212/wnl.43.11.2412-a 8232972

[3] Brent RJ. Advanced introduction to cost-benefit analysis. Cheltenham, UK: Edward Elgar; 2017.

[4] Brent RJ. Applied cost-benefit analysis, second edition. Cheltenham, UK: Edward Elgar; 2006.

[5] Morris JC. Clinical dementia rating: A reliable and valid diagnostic and staging measure for dementia of the Alzheimer type. Int Psychogeriatr. 1997; 9: 173–176. 9447441 10.1017/s1041610297004870

[6] Rikkert MG, Tona KD, Janssen L, Burns A, Lobo A, Robert P, Validity, reliability, and feasibility of clinical staging scales in dementia: A systematic review. Am J Alzheimers Dis Other Demen. 2011; 26: 357–365. 21914671 10.1177/1533317511418954 PMC10845587

[7] Weintraub S, Salmon D, Mercaldo N, Ferris S, Graff-Radford NR, Chui H, The Alzheimer’s disease centers’ Uniform Data Set (UDS): The neuropsychological test battery. Alzheimer Dis Assoc Disord. 2009; 23: 91–101. 19474567 10.1097/WAD.0b013e318191c7dd PMC2743984

[8] Beekly DL, Ramos ER, Lee WW, Deitrich ME, Jacka ME, Wu J, The National Alzheimer’s Coordinating Center (NACC) database: The Uniform Data Set. Alzheimer Dis Assoc Disord. 2007: 21:249–258. 17804958 10.1097/WAD.0b013e318142774e

[9] Morris JC, Weintraub S, Chui HC. The Uniform Data Set (UDS): Clinical and cognitive variables and descriptive data from Alzheimer disease centers. Alzheimer Dis Assoc Disord. 2006; 20: 210–216. 17132964 10.1097/01.wad.0000213865.09806.92

[10] Brent RJ. The value of a year’s general education for reducing the symptoms of dementia. Appl Econ. 2018; 50: 2812–2823. 29743729 10.1080/00036846.2017.1409420 PMC5937534

[11] Brent RJ. Estimating the monetary benefits of medicare eligibility for reducing the symptoms of dementia. Appl Econ. 2018; 50: 6327–6340. 30344332 10.1080/00036846.2018.1489519 PMC6195225

[12] Brent RJ. A CBA of hearing aids, including the benefits of reducing the symptoms of dementia. Appl Econ. 2019; 51: 3091–3103. 31631893 10.1080/00036846.2018.1564123 PMC6800143

[13] Stern Y. Cognitive reserve in ageing and Alzheimer’s disease. Lancet Neurol. 2012; 11: 1006–1012. 23079557 10.1016/S1474-4422(12)70191-6 PMC3507991

[14] Card D, Dobkin C, Maestas N. The impact of nearly universal health insurance coverage on health care utilization: Evidence from medicare. Am Econ Rev. 2008; 98: 2242–2258. 19079738 10.1257/aer.98.5.2242 PMC2600774

[15] Card D, Dobkin C, Maestas N. Does medicare save lives? Q J Econ. 2009; 124: 597–636. 19920880 10.1162/qjec.2009.124.2.597 PMC2777733

[16] Brent RJ. Cost-benefit analysis and health care evaluations, second edition, Cheltenham, UK: Edward Elgar; 2014.

[17] Zissimopoulos J, Crimmins E, St Clair P. The value of delaying Alzheimer’s disease onset. Forum Health Econ Policy. 2015; 18: 25–39. 10.1515/fhep-2014-0013 PMC4851168 27134606

[18] Hirth RA, Chernew ME, Miller E, Fendrick AM, Weissert WG. Willingness to pay for a quality adjusted life year: In search for a standard. Med Decis Making. 2000; 20: 332–342. 10929856 10.1177/0272989X0002000310

[19] Aldy JE, Viscusi WK. Adjusting the value of a statistical life for age and cohort effects. Rev Econ Stat. 2008; 90: 573–581.

[20] Plassman BL, Langa KM, Fisher GG, Heeringa SG, Weir DR, Ofstedal MB, Prevalence of dementia in the United States: The aging, demographics, and memory study. Neuroepidemiology. 2007; 29: 125–132. 17975326 10.1159/000109998 PMC2705925

[21] Petersen RC, Lopez O, Armstrong MJ, Getchius TSD, Ganguli M, Gloss D, Practice guideline update summary: Mild cognitive impairment. Neurology. 2018; 90: 1–10. doi:10.1212/WNL0000000000004826 [Epub ahead of print]. Table e-3 of online supplemental materials. Available at: links.lww.com/WNL/A34. Accessed January 11, 2018. PMC5772157 29282327

[22] Brookmeyer R, Abdalla N, Kawas CH, Corrada MM. Forecasting the prevalence of preclinical and clinical Alzheimer’s disease in the United States. Alzheimers Dement. 2018; 14: 121–129. 29233480 10.1016/j.jalz.2017.10.009 PMC5803316

[23] Condor RL Jr, Friesen C, Conder AA. Behavioral and complementary interventions for healthy neurocognitive aging. OBM Geriatrics. 2019; 3. doi: 10.21926/obm.geriatr.1901039.

[24] Gitlin LN, Hodgson RN, Jutkowitz E, Pizzi L. The cost-effectiveness of a nonpharmacologic intervention for individuals with dementia and family caregivers: The tailored activity program. Am J Geriatr Psychiatry. 2010; 18: 510–519. 20847903 10.1097/JGP.0b013e3181c37d13 PMC2938079

